# The impact of tacrolimus therapy on the outcomes of vernal keratoconjunctivitis: a systematic review and meta-analysis

**DOI:** 10.3389/fmed.2025.1542440

**Published:** 2025-07-30

**Authors:** Reem AlHuthail

**Affiliations:** Department of Ophthalmology, College of Medicine, Imam Mohammad Ibn Saud Islamic University (IMSIU), Riyadh, Saudi Arabia

**Keywords:** tacrolimus, vernal keratoconjunctivitis, ointment, suspension, VKC

## Abstract

**Background:**

Various preparations of tacrolimus have been implemented for patients with vernal keratoconjunctivitis (VKC). However, there is a lack of evidence regarding the safety and effectiveness of different dosages and forms of tacrolimus for patients with VKC.

**Objective:**

The present systematic review and meta-analysis evaluated the safety and effectiveness of various dosages and forms of tacrolimus for patients with VKC.

**Methods:**

The literature review was performed through 12 databases on 15 June 2024. All clinical studies comparing the outcomes of different dosages and tacrolimus preparations for VKC were included. Subgroup analysis was performed based on the dosages and formulations of tacrolimus.

**Results:**

The present meta-analysis included 17 articles, encompassing 832 patients with VKC. Of them, 421 patients received tacrolimus, while 411 patients were in the control group. Of the treated patients with tacrolimus, 66 were treated with tacrolimus ophthalmic suspension 0.1%, and 62 were treated with tacrolimus 0.1% ointment. Furthermore, 293 patients were treated with tacrolimus 0.03% ointment. There was a statistically significant (*p* = 0.02) difference between tacrolimus and the control group regarding the mean score for objective signs with SMD of −0.70 (95%CI:−1.28, −0.13). A statistically significant difference (*p* < 0.001) was observed between the tacrolimus ophthalmic suspension 0.1% and the control group with an SMD of −1.09 (95%CI:−1.59, −0.59). There was a significantly lower total subjective symptom score among patients treated with tacrolimus with an SMD of −0.86 (95%CI:−1.44, 0.28) and a probability value of 0.004. A statistically significant lower risk of treatment-related adverse events was revealed among patients treated with tacrolimus 0.03% ointment (*p* = 0.0002) with an RR of 0.16.

**Conclusion:**

Tacrolimus is an effective and safe therapeutic intervention for patients with VKC. It remarkably reduced the total score for objective signs and total subjective symptom score of VKC, with a relatively lower risk of treatment-related adverse events. The improvement of clinical manifestations was significantly associated with tacrolimus ophthalmic suspension 0.1%, while tacrolimus 0.03% ointment was associated with the lowest risk of treatment-related adverse events.

## Introduction

Vernal keratoconjunctivitis (VKC) is a chronic, bilateral yet often asymmetrical progressive disease of the cornea and conjunctiva, whose immuno-allergic etiopathogenesis is still being elucidated. It primarily affects young adults and children living in dry and warm climates, with an overall prevalence of 1% of ocular diseases (1, 2). Two variants of adult VKC have been developed based on the onset of the disease. Early-onset VKC begins in childhood and continues through adulthood, while late-onset VKC emerges after puberty. The hallmarks of VKC are conjunctival papillary inflammation and tissue remodeling (3, 4). The symptoms of the disease frequently occur seasonally and intensely, with the majority of patients experiencing photophobia, hyperemia, tearing, congestion, itching, and burning sensation. Although the disease is self-limiting and resolves around puberty, the worsening of the inflammatory phase of the VKC is bothersome. VKC can lead to sight-threatening sequels if treated inadequately. Blindness may result from corneal-related causes such as scarring, irregular astigmatism, ectasia, and limbal stem cell deficiency, as well as steroid-related side effects like cataract and glaucoma (5–7). The treatment of VKC depends on the frequency and severity of clinical manifestations and the duration of symptoms. The mainstay treatment of VKC is antihistamines, corticosteroids, immunosuppressive agents, non-steroidal anti-inflammatory drugs, and mast cell stabilizers (8, 9). Local drug administration may control the acute symptoms of VKC; however, the treatment of VKC is challenging, with no gold-standard therapy to control the recurrence and to prevent the progression of the disease (2, 10).

Immunomodulators are alternative therapies with potent anti-inflammatory effects and low adverse events. Tacrolimus is a macrolide immunosuppressant that has been extensively used in tissue transplants. Tacrolimus targets mainly CD4 + T lymphocytes, inhibiting calcineurin and suppressing interleukin 2, T helper 1 (Th1), and Th2 cytokines production. Furthermore, tacrolimus is a mast cell stabilizer, inhibiting histamine release and prostaglandin production (11, 12). The drug has been used in uveitis, corneal transplantation, and graft-versus-host disease. The topical application of tacrolimus significantly reduces the clinical manifestations of chronic allergic eye disorders with higher efficacy and low adverse events relative to corticosteroid therapy (13). The drug replaced corticosteroids for acute episodes and replaced other medications as a maintenance therapy for controlling VKC (11).

Various dosages and forms of tacrolimus have been reported in the literature. The high dosage of topical tacrolimus was associated with irritation, burning sensation, and epithelial keratitis. This may lead to low compliance among the pediatric population (14). There is a lack of evidence regarding the safety and effectiveness of different dosages and forms of tacrolimus for patients with VKC. Previously published reviews have revealed the therapeutic efficacy of tacrolimus for patients with VKC. However, these reviews have several limitations, with a limited number of included studies and heterogeneity due to the use of different preparations and strengths of tacrolimus (15, 16). This highlights the need for a more conclusive review evaluating tacrolimus’s effectiveness based on different dosages and formats. Such knowledge is essential to compel patients to use the most effective tacrolimus protocol to control the VKC course better. Therefore, the present systematic review and meta-analysis evaluated the safety and effectiveness of various dosages and forms of tacrolimus for patients with VKC.

## Methodology

The steps of the current systematic review and meta-analysis study followed the guidelines and the recommendations offered through the Cochrane Collaboration and Cochrane Handbook of Systematic Review and Meta-analysis (17) and based on the Preferred Reporting Items for Systematic Reviews and Meta-Analysis (PRISMA) guidelines (18) (Supplementary Table S1) (PROSPERO Number; CRD42024557297).

### Search methods

The literature review was performed through 12 databases on 15 June 2024. The following databases were searched using individualized search strings customized for each database: PubMed, ISI, Google Scholar, Scopus, NYAM, SIGLE, VHL, Clinical trials, mRCT, Cochrane Collaboration, EMBASE, and ICTRP. There were no limitations regarding age, gender, publication language, ethnicity, or study region. Citation tracking, cross-referencing, and reviewing the references of the eligible articles and previously published reviews were carried out to retrieve all possible relevant articles. The following keywords were used; “Tacrolimus,” “TCA,” “FK506,” “Vernal keratoconjunctivitis,” “VKC,” “Spring Catarrh.”

### Study selection

All clinical studies comparing the outcomes of different dosages and tacrolimus preparations for VKC were included. Non-comparative studies or those that did not report the outcomes of interest were excluded. Furthermore, studies in which data was inaccessible, guidelines, review articles, animal studies, case reports, comments, letters, editorials, posters, and book chapters were excluded. The articles retrieved from the screening process were exported to an Excel sheet after the initial removal of the duplicated reports using EndNote X9 (19). The title, abstract, and full-text screening processes were performed independently to disclose the potentially relevant articles that meet the eligibility criteria. The PRISMA flowchart documented the search process, screening, and the causes of article exclusion at each literature review step.

### Data extraction

The data were extracted in a well-organized Microsoft Excel sheet. The source-related data were extracted, including the title, study ID, study regions, study design, registration number, and study period. The methods-related data were extracted, including the eligibility criteria, diagnosis of VKC, previous therapies, dosage and formulations of tacrolimus, the dosages, and formulations of the control arm, grading of VKC, study endpoints, and follow-up periods. Baseline patients’ demographic characteristics were extracted, including sample size, age, body mass index (BMI), comorbidities, co-existing ocular diseases, and smoking history. The disease-related data were extracted, including the duration of the disease, type of VKC, severity of VKC, and symptoms and signs of VKC. The study endpoints were extracted, including total subjective symptom score, total objective symptom score, total objective sign score, and treatment failure. The data were extracted from the reported graphs in the Labcharoenwongs et al. using WebPlotDigitizer software (20).

### Study endpoint

#### Symptoms and signs

Total score for objective signs evaluated the palpebral conjunctiva, bulbar conjunctiva, limbus, and corneal involvement. The signs included hyperemia, edema, follicles, papillae, and giant papillae for the palpebral conjunctiva, hyperemia and chemosis for bulbar conjunctiva, and Trantas’ dot and edema for limbus signs. The corneal involvement was assessed using 4 grades: Normal = 0 Mild = 1+, moderate = 2+, Severe = 3+ (21). The total objective symptom score included hyperemia of bulbar and palpebral conjunctiva, papillae, giant papillae, and corneal infiltration (22). The analyzed articles reported the symptom scores differently for which the standardized mean difference (SMD) was used to standardize the results.

#### Treatment failure

Persistence of symptoms and signs of inflammation despite medication compliance.

#### Treatment-related adverse events

Any adverse event related to the medications including minor and major side effects of the medications.

#### Intraocular pressure

The intraocular pressure (IOP) was measured differently across the studies for which SMD standardize the results. We IOP was measured at the end of the follow-up visit.

#### Risk of bias and quality assessment

The risk of the bias of the included randomized clinical trials was evaluated based on the Cochrane Collaboration’s tool for assessing the risk of bias. This tool is composed of seven items; random sequence generation, allocation concealment (selection bias), blinding of participants and personnel performance bias, blinding of outcome assessment (detection bias), incomplete outcome data (attrition bias), selective reporting (reporting bias), and other possible causes of bias (23). The quality of the observational studies will be assessed using the National Institute of Health (NIH) quality assessment tool (24). The studies were assorted, based on this quality assessment, into good, fair, and bad when the score was >65%, 30–65, <30%, respectively. If the parameter was controlled, the domain was considered “Yes” and vice versa.

### Data analysis

Weighted mean difference (WMD) or standardized mean difference (SMD) was used to analyze the continuous variables. The SMD was used to evaluate the outcomes with different measurements or output values. Data reported in median and range, mean and range, mean and 95%confidence interval (CI) were converted to mean and standard deviation (SD) based on the equations of Hozo et al. (25). The risk ratio (RR) with 95% CI was used for analyzing dichotomous variables. The fixed-effect model was implemented when a fixed population effect size is assumed; otherwise, the random-effects model was used. Statistical heterogeneity was appreciated using Higgins *I^2^* statistic, at the value of > 50%, and the Cochrane Q (*Chi*^2^ test), at the value of *p* < 0.10 (26). The random-effects model was employed to account for this heterogeneity. Publication bias will be assumed in the presence of an asymmetrical funnel plot and based on Egger’s regression test (*p*-value < 0.10). Subgroup analysis was performed based on the dosages and formulations of tacrolimus. Data analysis was performed using Review Manager version 5.4 and Comprehensive Meta-Analysis v3 software (27, 28). The significant difference was established at the value of *p* < 0.05.

## Results

**Figure 1:**
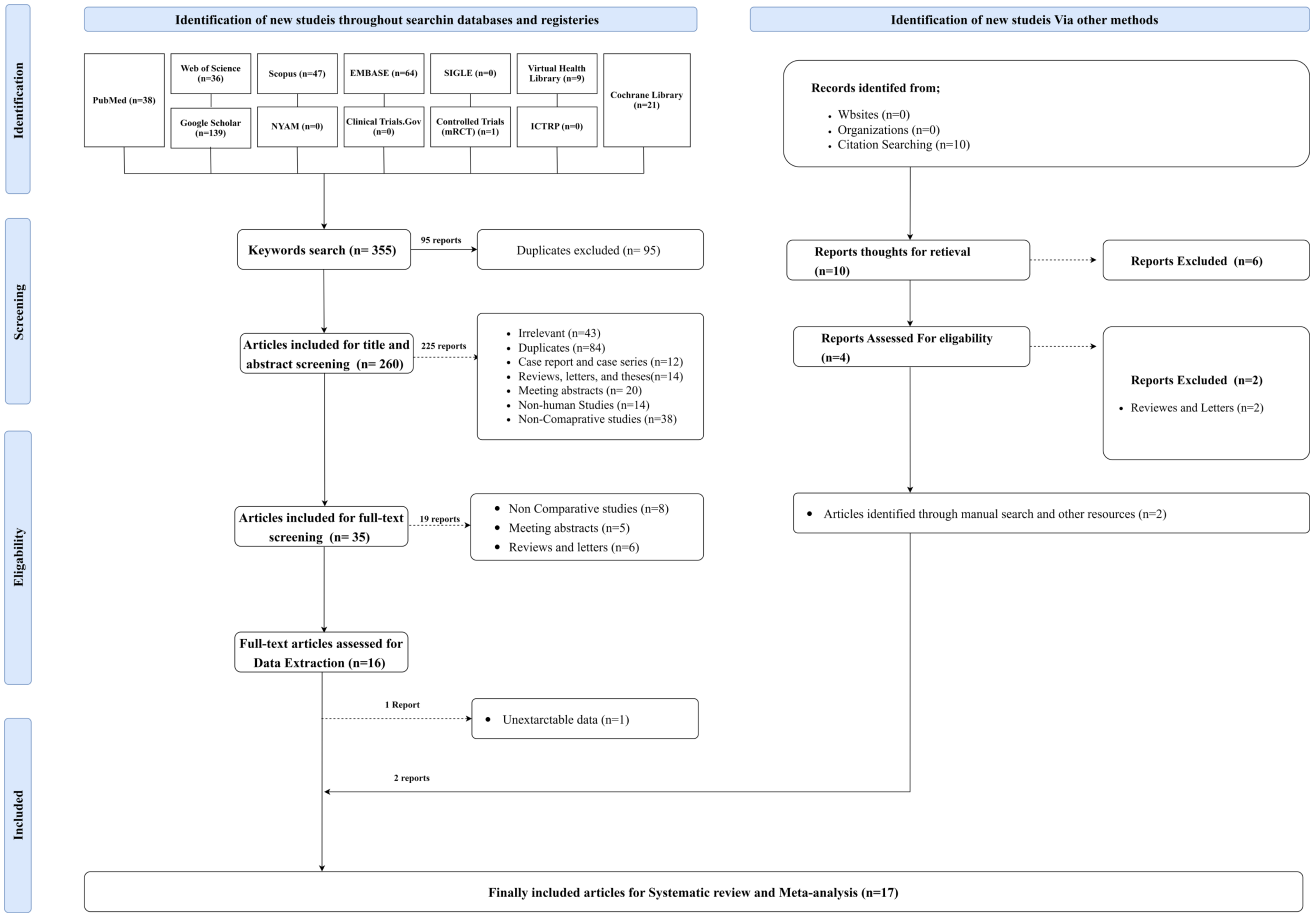
PRISMA flow chart showing the process of the literature search, title, abstract, and full text screening, systematic review, and meta-analysis.

Systematic searching of the literature revealed a total of 355 articles. Of them, 95 studies were duplicated, resulting in 260 reports included for title and abstract screening. Furthermore, 225 studies were excluded, and 35 were eligible for full-text screening. Sixteen articles were included for data extraction, one of which was excluded, and two were identified through the manual searching process. Seventeen articles were finally included for systematic review and meta-analysis (Figure 1).

### Demographic characteristics of the included studies

The present meta-analysis included 17 articles, encompassing 832 patients with VKC (29–45). Of them, 421 patients received tacrolimus, while 411 patients were in the control group. Of the treated patients with tacrolimus, 66 were treated with tacrolimus ophthalmic suspension 0.1%, and 62 were treated with tacrolimus 0.1% ointment. Furthermore, 293 patients were treated with tacrolimus 0.03% ointment. There were 12 articles of randomized controlled trials, while there were five observational studies. Nine articles used cyclosporine as the control group, while a placebo was used in two articles. There were 376 females and 96 males. The average age of the included patients ranged from 8 to 17.9 years among the tacrolimus group and from 7.12 to 15.2 years among the control group. There were 69 patients with limbal VKC and 129 patients with tarsal VKC. The average disease duration ranged from 12 months to 3.04 years among the tacrolimus group and 12 months to 3.21 years among the control group (Table 1).

**Table 1:** Demographic characteristics and quality assessment of the included studies.

Study ID		Study region	Study design	Study period	Intervention	Treatment protocol	Control	Sample size		Gender				Age (years)		Type of vernal keratoconjunctivitis						Disease duration		Quality assessment	
										Females		Males				Limbal		Tarsal		Mixed					
										Tacrolimus		Control		Tacrolimus	Control	Tacrolimus	Control	Tacrolimus	Control	Tacrolimus	Control	Tacrolimus	Control		
								Number	Number	Number	Number	Number	Number	Mean ± SD	Mean ± SD	Number	Number	Number	Number	Number	Number	Mean ± SD	Mean ± SD	%	Decision
1	Ohashi et al., 2010 (37)	Japan	RCT	February and September 2004	Tacrolimus ophthalmic suspension 0.1%	Twice daily for 4 weeks	Placebo	28	28	25	25	3	3	17.9 ± 9.1	15.2 ± 8.1	NR	NR	NR	NR	NR	NR	NR	NR	_____	_____
2	Pucci et al., 2015 (38)	Italy	RCT (Cross-over)	March 2008 to August 2010	Tacrolimus ophthalmic suspension 0.1%	1 drop 3 times daily in both eyes for 3 weeks	Cyclosporine eyedrops at 1%	30	30	24		6		9.05 ± 2.12		NR	NR	NR	NR	NR	NR	19.13 (7–31)^*^		_____	_____
3	Labcharoenwongs et al., 2012 (34)	Thailand	RCT	June 2003 to May 2005	Tacrolimus 0.1% ointment	Twice daily for 12 weeks	2% cyclosporine eye drops	12	12	11	12	1	0	10.14 ± 2.60	9.07 ± 2.50	1	4	7	8	4	0	2.93 ± 2.14	3.21 ± 2.51	_____	_____
4	Nivenius et al., 2007 (36)	Sweden	RCT (Cross-over)	January to April 2004	Tacrolimus 0.1% ointment	Twice daily for a 3-week period	clobetasone butyrate 0.05% ointment	20	20	NR	NR	NR	NR	10.14 ± 2.60		NR	NR	NR	NR	NR	NR	NR	NR		_____
5	Singla et al., 2017 (42)	India	RCT	NR	Tacrolimus 0.1% ointment	Twice daily for 6 weeks	Cyclosporine (2%)	30	26	NR	NR	NR	NR	8.33 ± 1.69	8.00 ± 1.60	23	16	5	8	2	2	16.47 ± 3.08	15.81 ± 2.55	_____	_____
6	Choudhary et al., 2019 (29)	India	RCT	May 2014 to May 2015	Tacrolimus 0.03% Ointment	NR	Cyclosporine (0.05%) ophthalmic eye drop	22	21	8	10	14	11	8.00 ± 0.81	7.57 ± 0.42	NR	NR	NR	NR	NR	NR	1.85 ± 0.20	1.71 ± 0.17	_____	_____
7	Eltagoury et al., 2022 (30)	Egypt	Non-randomized controlled clinical trial	NR	Tacrolimus 0.03% Ointment	Twice daily for 2 months	Standard anti-allergic medications	25	25	22	21	3	4	16.20 ± 5.10	16.48 ± 4.19	0	0	21	20	4	5	NR	NR	_____	_____
8	Gupta et al., 2021 (31)	India	Retrospective	1 January 2019 to 31 December 2020	Tacrolimus 0.03% Ointment	Three times a day	Interferon α-2b 1 MillionIU/mL	25	25	19	22	6	3	8.68 ± 2.53	7.92 ± 2.33	2	4	15	17	8	4	3.04 ± 1.21	2.96 ± 1.14	70%	Good
9	Heikal et al., 2020 (32)	Egypt	Prospective	October 2019 to February 2020	Tacrolimus 0.03% Ointment	Every 12 h in both eyes during the 12 weeks	Ciclosporine A eye drop (2%)	28	31	23	26	5	5	9.96 ± 4.16	10.83 ± 4.74	NR	NR	NR	NR	NR	NR	NR	NR	70%	Good
10	Kumari et al., 2018 (33)	India	RCT	March 2015-August 2015	Tacrolimus 0.03% Ointment	Twice daily for 6 weeks	Cyclosporine e/d (0.05%)	16	16	15	13	1	3	8.06 ± 1.94	7.12 ± 1.66	3	0	11	13	2	3	14	12	_____	_____
11	Malhotra et al., 2021 (35)	India	Prospective	NR	Tacrolimus 0.03% Ointment	Twice daily for 12 weeks	Cyclosporine 0.05%	19	19	NR	NR	NR	NR	NR	NR	NR	NR	NR	NR	NR	NR	NR	NR	70%	Good
12	Padmini et al., 2021 (41)	India	Prospective	December 2017 to February 2021	Tacrolimus 0.03% Ointment	Twice daily for 6 weeks	0.05% cyclosporin eye drops	36	36	30	32	6	4	NR	NR	NR	NR	NR	NR	NR	NR	NR	NR	70%	Good
13	Rathore et al., 2021 (40)	India	Cross-sectional study	February 2019 to July 2019	Tacrolimus 0.03% Ointment	NR	Olopatadine 0.2% eye drops	36	33	NR	NR	NR	NR	12.75 ± 5.54	8.88 ± 2.18	NR	NR	NR	NR	NR	NR	NR	NR	70%	Good
14	Suresha et al., 2023 (43)	India	RCT	NR	Tacrolimus 0.03% Ointment	Twice daily for 8 weeks	2% cyclosporine eye drops	29	27	24	14	5	13	NR	NR	NR	NR	NR	NR	NR	NR	NR	NR	_____	_____
15	Qin et al., 2018 (39)	China	RCT	NR	Tacrolimus ophthalmic suspension 0.1%	One drop/time, twice daily/1 drop/time, 4 times daily	tobramycin dexamethasone	29	27	NR	NR	NR	NR	12.56 ± 8.97		10	6	4	0	15	21	NR	NR	_____	_____
16	Zanjani et al., 2017 (44)	Iran	RCT	NR	0.005% tacrolimus	Two drops/time	interferonα-2b + placebo	28	27	NR	NR	NR	NR	NR	NR	NR	NR	NR	NR	NR	NR	NR	NR	_____	_____
17	Zhang et al., 2014 (45)	China	RCT	NR	0.005% tacrolimus	One drop/time, twice daily	placebo	8	8	NR	NR	NR	NR	NR	NR	NR	NR	NR	NR	NR	NR	NR	NR	_____	_____

RCT, Randomized controlled trial; SD, Standard deviation; NR, Non-reported. ^*^Data reported in the form of median and range.

### Risk of the bias and quality assessment

**Figure 2:**
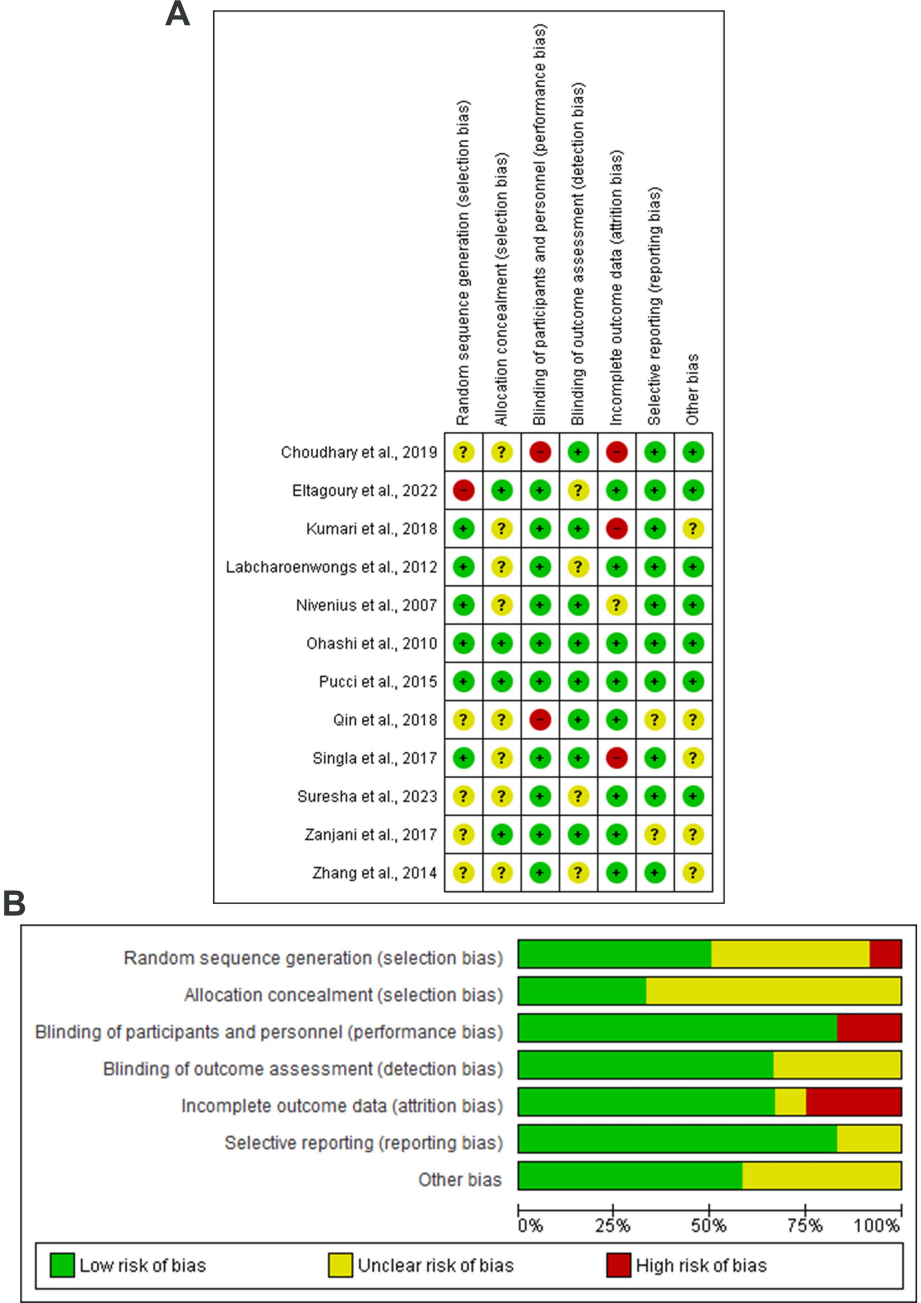
**(A)** Risk of bias graph, **(B)** Risk of bias summary: review authors’ judgments about each risk of bias item presented as percentages across all included studies.

Six articles showed a lower risk of random sequence generation bias (33, 34, 36–38, 42). Whereby four articles revealed a low risk of allocation concealment bias (30, 37, 38, 44), two articles showed a high risk of performance bias (29, 39). Eight articles showed a low risk of detection bias (29, 33, 36–42, 45), and three articles showed a high risk of attribution bias (29, 33, 42). Two studies showed unclear risk of reporting bias (39, 44). All the included studies showed good quality based on the NIH tool for quality assessment (Figure 2; Table 1).

### Study endpoints

#### Total score for objective signs

**Figure 3:**
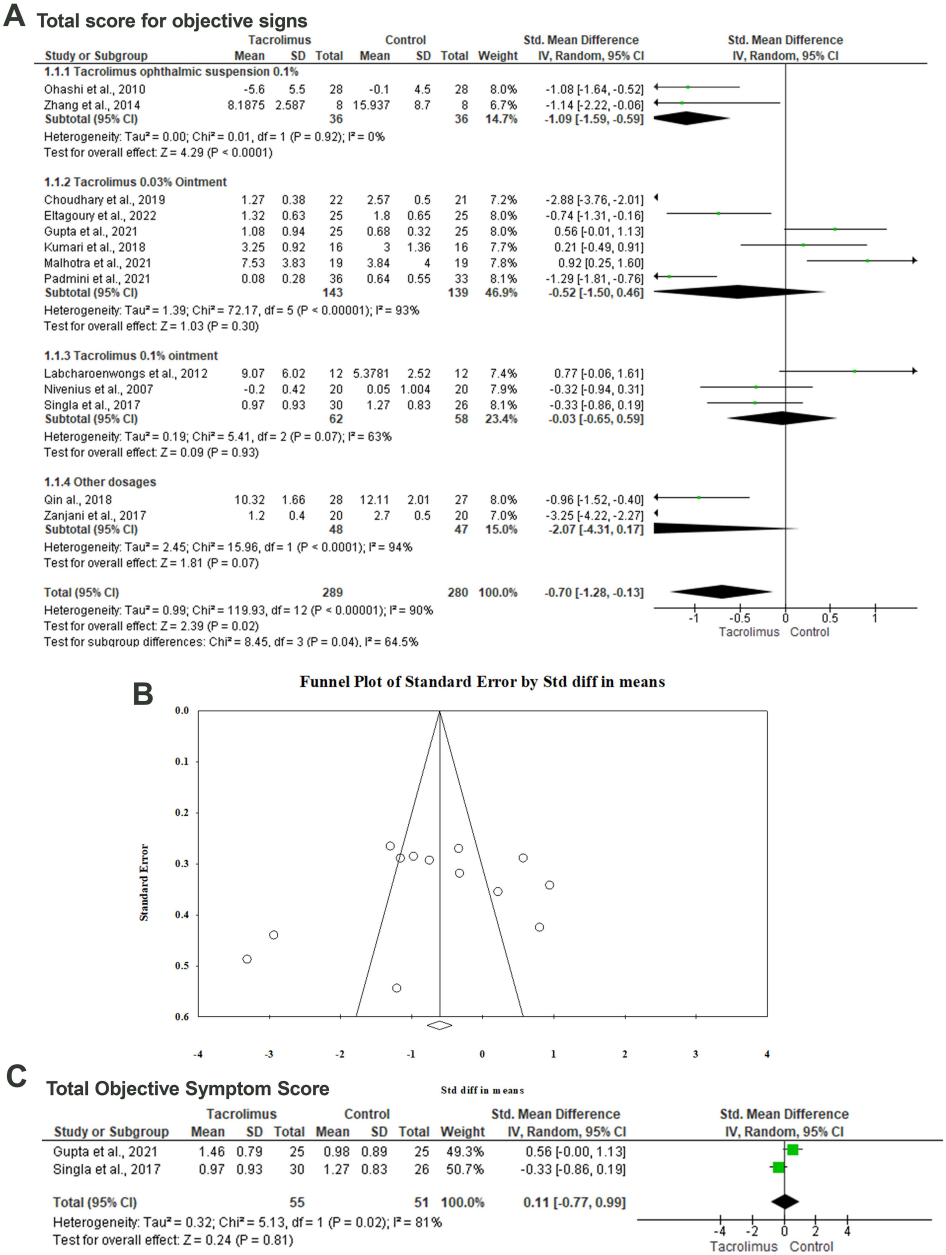
Forest plot of summary analysis of the **(A)** Standardized Mean Difference (SMD) and 95% CI of mean total score for objective signs between tacrolimus and control groups subgrouped by the dosages and formulations of tacrolimus. **(B)** Funnel plot showing the symmetrical distribution of the studies along the middle line. **(C)** Standardized Mean Difference (SMD) and 95% CI of the total objective symptom scores between tacrolimus and control groups. Size of the green squares is proportional to the statistical weight of each trial. The gray diamond represents the pooled point estimate. The positioning of both diamonds and squares (along with 95% CIs) beyond the vertical line (unit value) suggests a significant outcome (IV, inverse variance).

Thirteen articles included 569 patients evaluated the impact of different tacrolimus dosages on the total score for objective signs (29–31, 33, 34, 36, 37, 39–42, 44, 45). There was a statistically significant (*p* = 0.02) difference between tacrolimus and the control group with SMD of −0.70 (95%CI: −1.28, −0.13) in the random-effects model (I^2^ = 90%, *p* < 0.001). Pooling the data in the random-effects model (I^2^ = 0%, *p* = 0.92) revealed a statistically significant difference (*p* < 0.001) between the tacrolimus ophthalmic suspension 0.1% and control group with an SMD of −1.09 (95%CI: −1.59, −0.59). There was no statistically significant difference between the tacrolimus 0.1% ointment and the control group regarding the mean total score for objective signs (SMD: −0.52, 95%CI: −1.50, 0.46, *p* = 0.30) in the random-effects model (I^2^ = 63%, *p* = 0.007). In this respect, there was no statistically significant difference between tacrolimus 0.03% ointment and the control group with an SMD of −0.03 and 95CI% ranging from −0.65 to 0.59 (*p* = 0.93). No evidence of publication bias was detected based on the results of Egger’s regression test (Intercept = −3.855, *p* = 0.4) (Figures 3A,B).

#### Total objective symptom score

The difference between tacrolimus and control groups regarding the mean total objective symptom scores was reported in two articles among 106 patients (31, 42). Metaanalyzing the data in the random-effects model (I^2^ = 81%, *p* = 0.02) revealed no statistically significant difference between both groups with an SMD of 0.11 (95%CI: −0.77, 0.99, *p* = 0.81) (Figure 3C).

#### Total subjective symptom score

**Figure 4:**
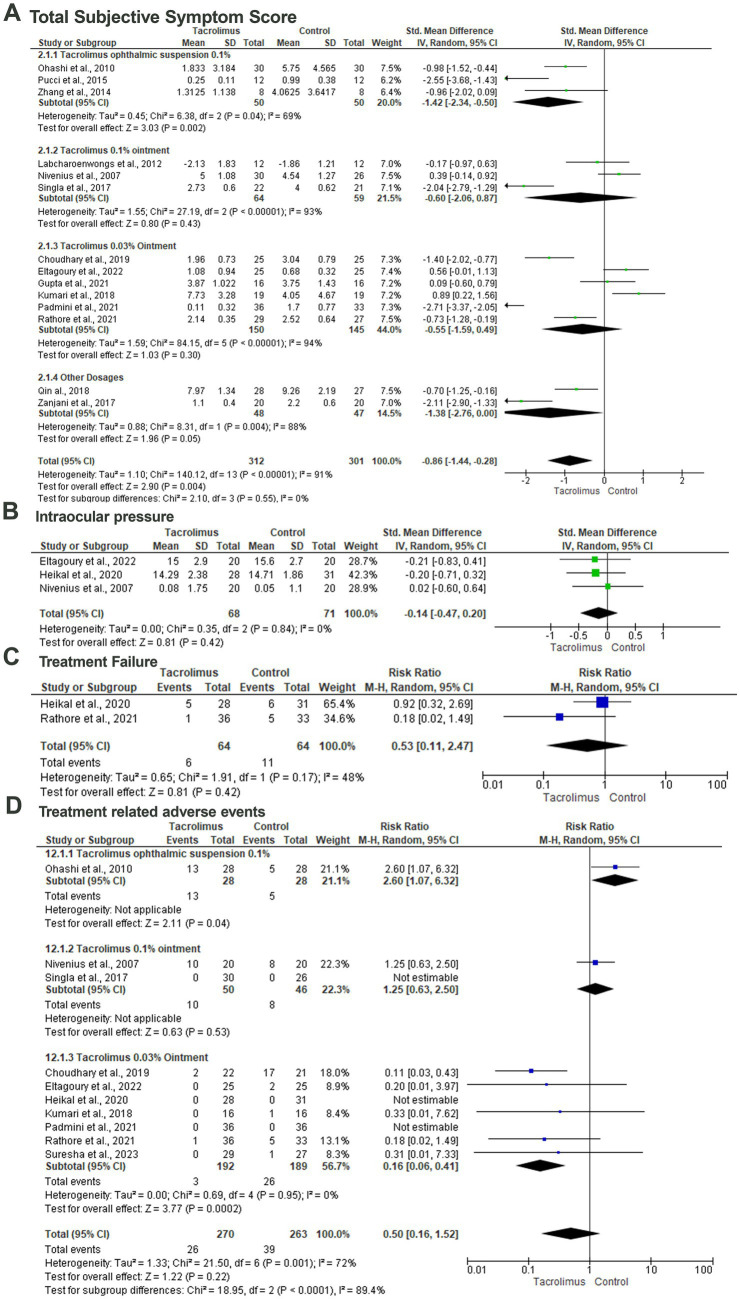
Forest plot of summary analysis of the **(A)** Standardized Mean Difference (SMD) and 95% CI of mean total subjective symptom score between tacrolimus and control groups subgrouped by the dosages and formulations of tacrolimus. **(B)** Standardized Mean Difference (SMD) and 95% CI of the mean intraocular pressure between tacrolimus and control groups. **(C)** Risk ratio and 95% CI of the risk of treatment failure between tacrolimus and control groups. **(D)** Risk ratio and 95% CI of the risk of treatment-related adverse events between tacrolimus and control groups. Size of the green or blue squares is proportional to the statistical weight of each trial. The gray diamond represents the pooled point estimate. The positioning of both diamonds and squares (along with 95% CIs) beyond the vertical line (unit value) suggests a significant outcome (IV, inverse variance).

Fourteen articles included 613 patients evaluated the difference between tacrolimus and control groups regarding the mean total subjective symptom score (29–31, 34–42, 44, 45). In the random-effects model (I^2^ = 91%, *p* < 0.001), there was a statistically significant lower total subjective symptom score among patients treated with tacrolimus with an SMD of −0.86 (95%CI: −1.44, 0.28) and probability value of 0.004. Subgroup analysis based on the dosage and form of tacrolimus revealed a statistically significant lower mean total subjective symptom score among patients treated with tacrolimus ophthalmic suspension 0.1% (SMD; −1.42; 95%CI: −2.34, −0.50; *p* = 0.002) in the random-effects model (I^2^ = 69%, *p* = 0.04). There was no statistically significant difference between tacrolimus 0.1% ointment and the control group regarding the mean total subjective symptom score with an SMD of −0.60 (95%CI: −2.06, 0.87; *p* = 0.43). In the random-effects model (I^2^ = 94%, *p* < 0.001), there was no statistically significant difference between tacrolimus 0.03% ointment and the control group (SMD; −0.55, 95%CI: −1.59, 0.49, *p* = 0.30) (Figure 4A).

### Intraocular pressure

Three studies included 139 patients evaluated the difference in intraocular pressure between the tacrolimus and control group within three studies (30, 32, 36). Pooling the data revealed no statistically significant difference between the tacrolimus and the control group with an SMD of −0.14 and 95%CI of −0.47 to 0.20 and probability value of 0.42 in the random-effects model (I^2^ = 0%, *p* = 0.48) (Figure 4B).

### Treatment failure

The risk of treatment failure was evaluated among 17 patients within two studies (32, 40). In the random-effects model (I^2^ = 48%, *p* = 0.17), there was no statistically significant difference between the tacrolimus and control groups with a RR of 0.53 (95%CI: 0.11, 2.47) and probability value of 0.42 (Figure 4C).

### Treatment-related adverse events

Ten articles included 533 patients evaluated the treatment-related adverse events with tacrolimus (29, 30, 32, 33, 36, 37, 40–43). Pooling the data in the random-effects model (I^2^ = 72%, *p* = 0.001) revealed no statistically significant difference between tacrolimus and control groups with a RR of 0.50 (95%CI: 016, 1.52) and probability value of 0.22. Subgroup analysis based on the dosage of the tacrolimus revealed a statistically significant lower risk of treatment-related adverse events among patients treated with tacrolimus 0.03% ointment (*p* = 0.0002) with a RR of 0.16 and 95%CI% ranged from 0.06 to 0.41 in the random-effects model (I^2^ = 0%, *p* = 0.95) (Figure 4D).

## Discussion

VKC is a severe form of ocular allergy which is associated with considerable morbidities. The disease can cause visual loss and primarily begins in children aged between 2 and 10 years old. There has been a controversial result in the literature regarding the impact of different concentrations and tacrolimus preparations on patients with VKC (46). The present meta-analysis revealed the therapeutic efficacy of tacrolimus in treating patients with VKC. Particularly, tacrolimus significantly improved the total score for objective signs and total subjective symptom score with a relatively lower risk of treatment-related adverse events. The tacrolimus ophthalmic suspension 0.1% achieved statistically significant results compared to the control group, relative to tacrolimus 0.03% ointment and tacrolimus 0.1% ointment. The risk of treatment-related adverse events was reduced by approximately 50% among the tacrolimus group. This risk was reduced more remarkably among patients treated with tacrolimus 0.03% ointment. These findings revealed the excellent efficacy of tacrolimus ophthalmic suspension 0.1% in treating patients with VKC, promoting the potentiality of using this formulation to enhance local tolerability.

The present meta-analysis revealed a significant reduction in symptoms and signs of VKC among patients treated with tacrolimus, particularly tacrolimus ophthalmic suspension 0.1%. These findings were consistent with Zhao et al. (16) who revealed the efficacy of tacrolimus among patients with VKC, reducing congestion, itching, tearing, foreign body sensation, and objective patient signs. Chandra et al. (15) review revealed a significant reduction in symptoms and signs of VKC with topical tacrolimus therapy. Contrary to these findings, Roumeau et al. (47) revealed a similar efficacy of tacrolimus and cyclosporine among patients with severe VKC. Furthermore, they revealed that this efficacy does not differ based on the concentration of tacrolimus, highlighting that a low dosage may be sufficient. The local tolerance of these formulations is mainly concentration-dependent. Fazri et al. (48) revealed that tacrolimus effectively treated patients with VKC, particularly for patients with corticosteroid-refractory VKC.

The present meta-analysis revealed a lower risk of treatment-related adverse events, particularly among patients treated with tacrolimus 0.03% ointment. Tacrolimus is a hydrophobic substance that is unstable at clinically effective concentrations. The aqueous preparation of tacrolimus needed to be prepared in olive oil, castor oil, and dextrin. Conversely, these preparations were associated with redness, burning sensation, itching, and epithelial keratitis. The penetration of the corneal epithelium is difficult due to its unique properties and large molecular size. The dermal ointments were used to attempt such adverse events, which proposed to achieve tolerability and less toxicity (49, 50). Tacrolimus in ointment may have a beneficial effect due to the long-standing effect. Consistent with our findings, Akbari et al. (48) review revealed that topical tacrolimus 0.05% is an effective and safe agent to treat refractory VKC with no systemic or ocular adverse effects.

The present meta-analysis revealed tacrolimus’s functional and safety outcomes among patients with VKC. The study included the largest cohort in the literature, assessing the outcomes of tacrolimus in different dosages and formulations. Conversely, some limitations should be considered while interpreting the resulting evidence. While most eligible studies were randomized controlled trials, some were observational, which confer a substantial risk of information bias. Subsequently, there was significant statistical and methodological heterogeneity between the analyzed articles. This heterogeneity may be attributed to the considerable variation between the analyzed articles regarding the recruitment criteria, sample sizes, follow-up period, treatment protocol, control arm, disease severity, study outcomes, and demographic characteristics of the included patients. The resulting statistical heterogeneity was mitigated by applying the random-effects model and doing subgroup analysis. The wide variations of the control group limited the capability to conduct network meta-analysis. Further randomized controlled trials with adequate samples and prolonged follow-up periods are necessary to mitigate the potential limitations of the analyzed studies.

## Conclusion

Tacrolimus is an effective and safe therapeutic intervention for patients with VKC. It remarkably reduced the total score for objective signs and total subjective symptom score of VKC, with a relatively lower risk of treatment-related adverse events. The improvement of clinical manifestations was significantly associated with applying tacrolimus ophthalmic suspension 0.1%, while tacrolimus 0.03% ointment was associated with the lowest risk of treatment-related adverse events.

## Data Availability

The original contributions presented in the study are included in the article/Supplementary material, further inquiries can be directed to the corresponding author.
